# Virology, epidemiology, transmissions, diagnostic tests, prophylaxis and treatments of human Mpox: Saudi Arabia perspective

**DOI:** 10.3389/fcimb.2025.1530900

**Published:** 2025-02-28

**Authors:** Almonther Abdullah Hershan

**Affiliations:** Department of Basic Medical Sciences, College of Medicine, The University of Jeddah, Jeddah, Saudi Arabia

**Keywords:** Mpox, Saudi Arabia, virology, prophylaxis, vaccines, therapeutics

## Abstract

Mpox (Monkeypox) is a highly contagious viral disease that can be transmitted from animal-to-human or human-to-human through intimate contact, Mpox is caused by the monkeypox virus (MPXV), which is an enveloped double-stranded DNA that belongs to the genus *Orthopoxvirus, Poxviridae* family, and subfamily *Chordopoxvirinae.* Mpox cases were previously only reported in West and Central Africa, however in recent times non-endemic countries including Saudi Arabia (SA) also reported confirmed Mpox cases. The first laboratory-confirmed human Mpox case in SA was reported on 14 July 2022, since then a number of confirmed Mpox cases have been reported by WHO in SA. These confirmed Mpox cases in SA were observed among individuals with a history of visiting European Union countries. SA is not only at risk of importation of Mpox cases owing to travel to such countries, but also there are various other risk factors including geographic proximity to the African continent, trade in exotic animals, and massive inflow of tourists. Therefore, government health authorities of SA should continue to collaborate with various international health organizations including WHO to prevent, manage or monitor potential health risks at most of the entry points in SA including highways, seaports, and airports by ensuring adherence to hygiene protocols, vaccinations, and health screenings. There are a range of diagnostic tests are currently available that can be used in SA to confirm Mpox infections, including real-time PCR, loop-mediated isothermal amplification, serological testing, clustered regularly interspaced short palindromic repeat-CRISPR-associated protein (CRISPR-Cas)-based systems, whole-genome sequencing, electron microscopy, and virus isolation and culture. There is no approved treatment specifically for Mpox, however multiple approved antiviral agents for smallpox treatment were found to be useful in Mpox treatment and in the management of Mpox outbreaks, such as- trifluridine, brincidofovir, tecovirimat, and cidofovir. The aim of this review is to provide valuable insights regarding virology, pathogenesis, epidemiology, transmissions, clinical presentation, diagnostic tests, prophylactic measures and therapeutic options of Mpox from SA perspective. Moreover, a side-by-side discussion on the global trend and scenarios of Mpox has been provided for comparison and further improvement in measures against Mpox in SA.

## 1 Introduction

Mpox (previously known as monkeypox) is a viral infectious disease caused by the Mpox virus (MPXV), which is an enveloped double-stranded DNA virus that belongs to the *Poxviridae* family of the genus *Orthopoxvirus* (Mohapatra et al., 2024). MPXV infects a range of animals including non-human primates, such as monkeys, dogs, squirrels, rodents, and others. In 1958, Mpox was first identified in monkeys reserved for research in an animal facility in Denmark (Parker and Buller, 2013). Humans are accidental hosts of MPXV and the first human Mpox case was documented in the Democratic Republic of the Congo (DRC) in 1970 (Mohapatra et al., 2024). Mpox cases were initially common in the African countries including DRC, Sierra Leone, Liberia, Central African Republic, South Sudan, Gabon, Nigeria, and Cameroon (Suvvari et al., 2023). However, an increased number of human cases of Mpox was reported in all 5 continents in 2022. Both U.S. Centers for Disease Control and Prevention (CDC) and World Health Organization (WHO) have classified Mpox as an emerging disease because of its rapid dissemination and high infectivity (Islam et al., 2023). In addition, between July 2022 and May 2023, WHO declared Mpox as a Public Health Emergency of International Concern (Kuehn et al., 2024). From 1 January 2022 to 11 October 2024, the WHO has already reported 106,310 laboratory-confirmed Mpox cases and 234 deaths in 123 countries (World Health Organization, 2024a).

Over the past four decades, multiple factors have contributed in the increased incidences of the Mpox outbreak. Smallpox immunization program discontinuance is one such factor. It has been demonstrated that the smallpox vaccine can provide up to around 85% effectiveness in preventing Mpox. Extensive intake of animal meat as a protein source particularly in the civil war and poverty-stricken regions, where the animal is a potential reservoir of MPXV. Other factors that are playing a role in increasing Mpox cases include elevated exposure to reservoir animals, environmental and ecological conditions (for example- clearance of tropical rainforests), the ease of transboundary travel, and the growing human population worldwide (Dhawan et al., 2022; Fahrni et al., 2022; Priyanka et al., 2023a, 2023b; Choudhary et al., 2024; Mohapatra et al., 2024). Interestingly, various meteorological factors also play a major role in Mpox cases, where temperature is found to be positively linked with Mpox cases and wind speed is negatively linked (Islam et al., 2022b, 2024). There is also a link between Mpox cases and obesity, where increased occurrence of Mpox has been observed in obese individuals (Hasan et al., 2023).

Mpox was previously only reported in West and Central Africa, however in recent times non-endemic countries also reported confirmed Mpox cases including the Saudi Arabia (SA) and its neighboring countries (Assiri et al., 2024). The first laboratory-confirmed human Mpox case in SA was reported on 14 July 2022 and a total of 8 confirmed Mpox cases were reported by WHO as of 30 June 2024 (Assiri et al., 2023; World Health Organization, 2024a). In SA, confirmed Mpox cases were observed among individuals with a history of visiting European Union countries. SA is not only at risk of importation of Mpox cases owing to travel to such countries, but also there are various other risk factors including geographic proximity to the African continent, trade in exotic animals, and massive inflow of tourists. Moreover, unlike earlier outbreaks, it has been observed that there is also a change in the nature of Mpox epidemiology and an increased number of older and middle-aged adults are being affected by this infectious disease (Bunge et al., 2022). Since WHO has declared Mpox as a health emergency, therefore the Ministry of Health (MOH) in SA has developed some guidelines and preventive measures to prevent the spread and transmission of MPXV including targeted use of the 2^nd^ or 3^rd^-generation Mpox or smallpox vaccines, the use of typical polymerase chain reaction (PCR) techniques, timely screening with nucleic acid amplification testing (NAAT), and tracing and isolating Mpox affected individuals (Alshahrani et al., 2022b). This review aims to provide valuable insights regarding virology, pathogenesis, epidemiology, transmissions, clinical presentation, diagnostic tests, prophylactic measures and therapeutic options of Mpox from SA perspective. Moreover, a side-by-side discussion on the global trend and scenarios of Mpox have been provided for comparison and further improvement in measures against Mpox in SA.

## 2 Virology of MPXV

### 2.1 Classification

MPXV is an enveloped double-stranded DNA that belongs to the genus *Orthopoxvirus, Poxviridae* family, and subfamily *Chordopoxvirinae.* MPXV is closely related to cowpox virus, vaccinia virus (VACV), and variola virus (Bunge et al., 2022). Clade I or Congo Basin clade and Clade II or West African clade are the 2 distinct MPXV phylogenetic clades. There are 2 subclasses of Clade II including Clade IIa and Clade IIb. In general, the genomic sequences of Clade I and Clade II derived MPXV strains show an overall 99% identity of nucleotides within the same region, while 95% nucleic acid identity across different geographical clusters (Liang et al., 2022).

### 2.2 Genomic organization and morphology of MPXV

**Figure 1:**
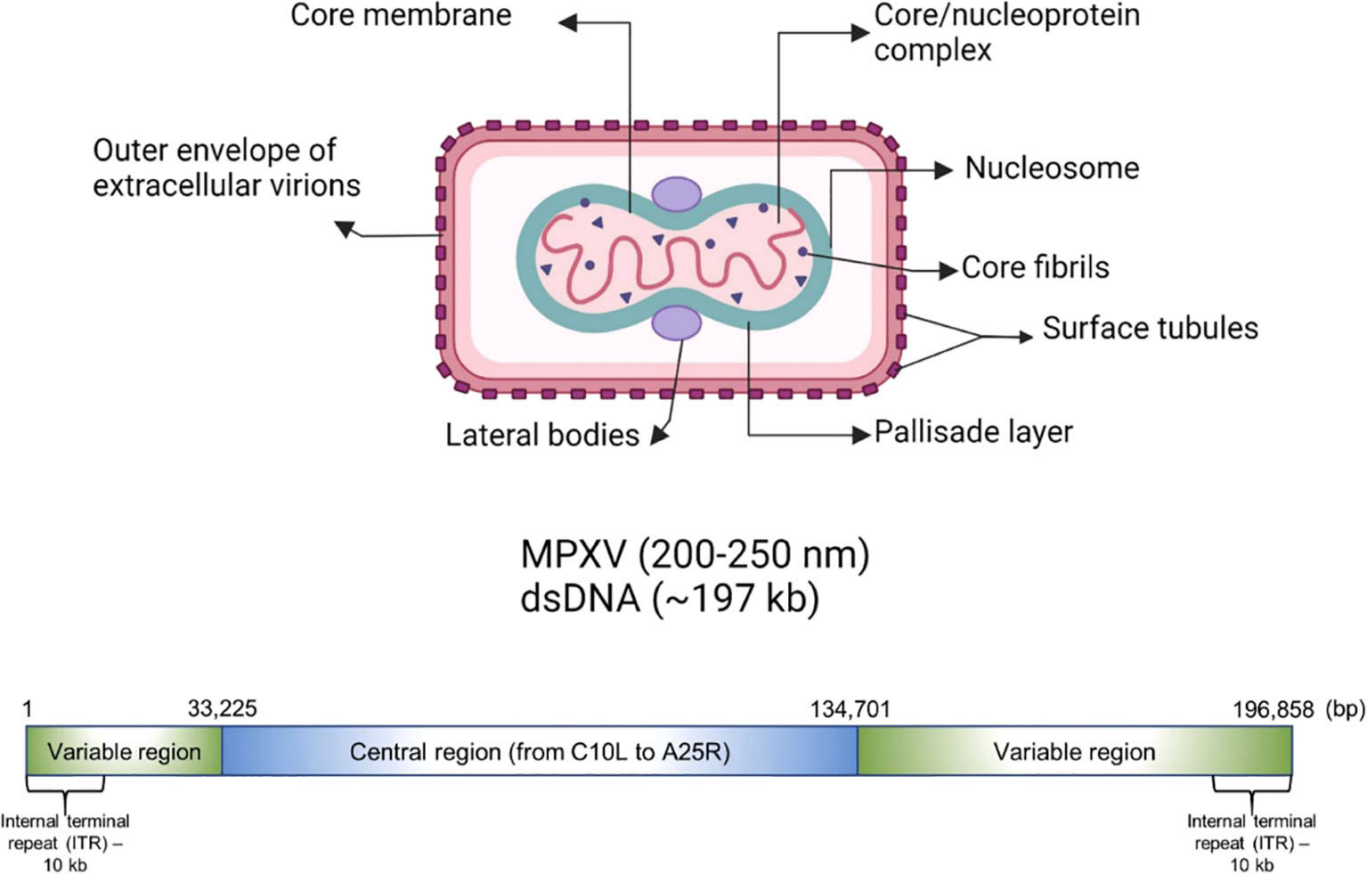
Genomic organization and morphology of MPXV. Reproduced with permission from Elsevier, (Karagoz et al., 2023).

The genome of MPXV is around 197,000 kb and possesses over 190 open reading frames (ORFs), with inverted terminal repeats flanking the terminal ends (**Figure 1**). A minimum of 90 ORFs are essential for the poxvirus replication and morphogenesis. A number of non-essential ORFs are supposed to have a significant contribution in the alterations in poxvirus host tropism, pathogenesis, and immunomodulation, while the functions of most of the ORFs are yet to be characterized. The virions of MPXV have an average size of 280 nm × 220 nm and they are either oval or barrel-shaped. A unique dumbbell-shaped nucleoprotein core is present in poxvirus mature particles and they contain a large genome composed of double-stranded linear DNA. Moreover, MPXV virions possess virus-encoded DNA-dependent RNA polymerases and more than 30 viral structural and membrane proteins (Yu et al., 2023). Essential orthopoxvirus (OPV) genes are found in the central portion of the MPXV genome. Nonetheless, as compared to the genomes of other OPVs, a small number of ORFs are either truncated or lost in the genome of MPXV. Multiple disrupted ORFs encoding genes associated with immune evasion have been reported in Clade II, where these mutations might be responsible for the lesser virulence of Clade II in comparison with Clade I (Reynolds and Damon, 2012).

The extracellular enveloped virus (EEV) and intracellular mature virus (IMV) are the 2 different infectious MPXV forms. These infectious forms are also different in terms of cell-infecting mechanisms and surface glycoproteins. IMV is released during cell lysis, while EEV is accountable for early dispersal. The major structural difference between EEV and IMV is that the additional outermost membrane layer is not present in IMV. There is also a difference in the quantities of viral proteins incorporated into the 2 types of virions (Yu et al., 2023). MPXV replicates through a complex process, however it is generally thought to be similar to that of other OPVs. The entry receptors of MPXV are yet to be fully discovered. Nonetheless, it is supposed that the entry of MPXV is reliant on host cell type as well as viral strain as well as involves many surface receptors including chondroitin sulfate or heparan sulfate (Pickup, 2015).

## 3 Epidemiology of MPXV

### 3.1 Global epidemiology

During the time of initial identification, 282 human cases were reported in Zaire between 1980 and 1985. The age range of the affected individuals was from 1 month to 69 years, where 90% of them were under 15 years of age. However, there was an 11% mortality rate in unvaccinated patients along with an increased rate in children (15%), while no death was reported in vaccinated individuals (Karagoz et al., 2023). A study also reported an increased case fatality rate among the young children as compared to general population (Islam et al., 2022a). Immunization against smallpox typically provides protection against Mpox, however smallpox eradication and consequent reduction in the vaccination campaign hindered the cross-protective immunity against Mpox. Lack of proper MPXV reporting from rural Africa leads to an underestimated threatening potential of MPXV. Following an under-reported period of 39 years in Nigeria’s Bayelsa State, MPXV re-emerged in 2017, which was then supposedly exported to Israel and other countries through travelers. Subsequently, the number of Mpox cases were increased in 2018 and 2019. A number of factors played a role in the MPXV outbreak outside Africa including direct contact with infected monkeys, travelling, shipping, and importation, or susceptible populations with a risk of contracting Mpox. Moreover, smallpox vaccine cessation might also have contributed in increased MPXV transmission from human-to-human. MPXV outbreaks outside Africa indicate the global connection of Mpox infection. Thus, measures are needed to promote diagnosis and monitoring to find out the fluctuating epidemiology of Mpox (Bunge et al., 2022). Mpox not only affected Africa, but also developed countries. In the U.S., two Mpox cases were observed the persons who returned from Nigeria to Texas in July 2021. On May 6, 2022, a British man was also found to be Mpox infected, after his visit to Nigeria. As of September 24, 2024, more than 106 thousand confirmed cases, including in Europe and North America, have been reported. Mpox is commonly detected in western and central Africa; however Mpox cases have been reported in developed countries as well, which indicates that MPXV is spreading globally. According to WHO definitions, a suspected case is someone who is a contact of a confirmed or probable Mpox case in the 21 days before the onset of symptoms or signs including fatigue, profound weakness, back pain, myalgia, headache, acute onset of fever (>38.5°C) (World Health Organization, 2024b). On the other hand, a probable case is someone who exhibits lymphadenopathy, mucosal lesions, unexplained acute skin rash, and has a positive test result for orthopoxviral infection, had several and/or casual sexual partners in the 3 weeks before onset of symptoms, and has an epidemiological link to a confirmed or probable case of Mpox in the 21 days before onset of symptoms (World Health Organization, 2024b). A confirmed Mpox case requires laboratory-confirmed Mpox virus by detection of unique Mpox DNA sequences through real-time PCR (RT-PCR) and/or sequencing (World Health Organization, 2024b). All MPXV-infected cases took place because of travelling to Africa or through animal shipping. In the U.S. alone, around 29,980 probable and confirmed cases and 80 mortalities have been reported. Collectively, these findings suggest that Mpox is a global threat, which further indicates the necessity of developing a strategic plan to avert Mpox from becoming a pandemic (Karagoz et al., 2023).

### 3.2 Saudi Arabia epidemiology

Limited data is available regarding Mpox epidemiology in SA. Assiri et al (Assiri et al., 2024). investigated the continued Mpox occurrence in SA after removal of public health emergency (between July 2022 and May 2023) of Mpox by WHO (Assiri et al., 2024). The researchers investigated 381 cases of Mpox in SA from May to September 2023. They found that 91.1% of the Mpox-affected individuals are males with an average age of 32.4 (± 8.3) years. Out of 381 individuals, 379 (99.5%) individuals were not associated with secondary cases, 277 (72.7%) individuals refused engagement in extra-marital sex, and 356 (93.4%) of them did not report travelling. Headache was experienced by 314 (82.4%) individuals, while fever was present in 371 (97.4%) cases. In terms of lesions, the most commonly affected areas include the mouth (160 cases, 42%), face (198 cases, 52%), genitals (206 cases, 54%), and palms and soles (297 cases, 78%). All of the genotyped samples were found to be subclade IIb (Assiri et al., 2024). In a different study at King Saud Medical City (KSMC), Dar et al. provided a detailed report of 16 confirmed Mpox cases detected in Riyadh, SA between June and September 2023. The diagnosis and management of confirmed and suspected cases were carried out by following guidelines developed by Saudi Public Health Authority (Weqaya) (Ministry of Health, 2023), Among these 16 laboratory-confirmed cases, 2 were Saudi, 9 Pakistani, 2 Bangladeshi, 2 Indian and one Yemini. In addition, 14 out of 16 affected individuals are migrant workers in SA for over 1 year. The average age of the confirmed individuals was 33.9 years and 15 of them were males. No mortality or substantial complications were reported among the patients (Dar et al., 2023).

## 4 Pathogenesis of MPXV

**Figure 2:**
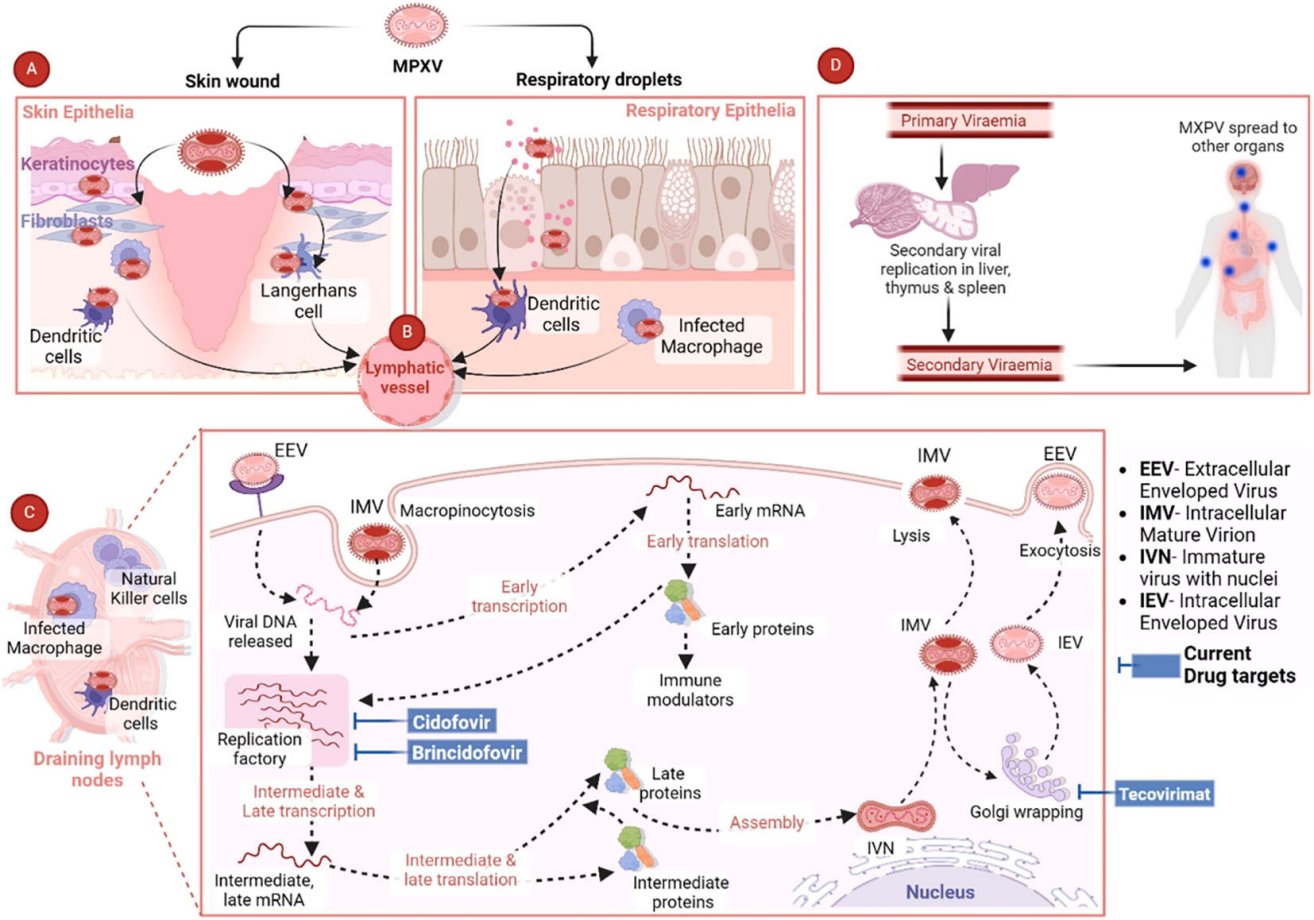
Pathogenesis of MPXV and current antiviral drug targets. Reproduced with permission from Elsevier, (Singh et al., 2024). **(A)** The figure shows the MPXV interaction with respiratory epithelia or skin. **(B)** After the initial MPXV-caused infection, the virus is transported via the lymphatic pathways to the draining lymph nodes. **(C)** MPXV mainly uses the micropinocytosis process to enter the host cells, which includes engulfing MPXV via the host cell membrane to generate an internal cyst. **(D)** MPXV can cause infection in various major organs including the thymus, spleen, and liver, where secondary viral replication can occur. Then the secondary viremia can result in the dissemination of MPXV in various distant organs including the intestines, kidneys, and lungs. The final spread can result in elevated disease severity and extensive organ involvement (Singh et al., 2024).

Mpox is a highly contagious disease that can be transmitted from animal-to-human or human-to-human through close contact of any kind, which is regarded as the first step in the pathogenesis and pathogenicity of MPXV. There are several ways through which MPXV can be transmitted from person to person. Different modes of transmission have been further discussed in the “Transmission of MPXV” section. The entry of MPXV particles into host cells is the first step of viral pathogenesis in humans. A number of studies have been carried out to elucidate the interaction between MPXV and response of host cells, which suggests significant genetic diversity in the clade-specific genes across Clade I and II. Indeed, this variation plays a role in the pathogenicity showed by these entities. MPXV genome is composed of several genes that are responsible for encoding host-response modifier proteins. Mpox inhibitor of complement enzymes (MOPICE) are considered as the virulence factors of MPXV. It has been observed that the MOPICE protein has a significant contribution in elevating pathogenicity in the case of Clade I, while Clade II is lacking this protein. In the human body, MPXV can be introduced through various routes, such as intradermal routes, respiratory routes, or during sexual intercourse (Choudhary et al., 2022a). On August 17, 2024, The Public Health Authority (Weqaya) released a statement that no Clade I case has been detected in SA (Abid, 2024). The MPXV multiplies at the site of inoculation and then spreads into blood, lymph nodes, bone marrow, spleen, and tonsils, which eventually results in primary viremia and MPXV transportation into skin and testes (Hatmal et al., 2022). It has been revealed by *in vivo* studies that the primary sites of MPXV replication include lymphoid tissues in the throat and neck. Following this primary MPXV replication, it further infects the liver and spleen (Lum et al., 2022). MPXV also infects Langerhans cells, dendrites, and macrophages (**Figure 2**), which eventually results in inflammation of keratinocytes and fibroblasts (Cann et al., 2013). MPXV enters or invades the host cells either through macropinocytosis or a fusion mechanism. Indeed, the MPXV entry and release mechanisms are complicated owing to the co-occurrence of two different viral forms including IMV and EEV, which are enclosed by diverse lipid bilayers and show characteristic surface proteins (Saied et al., 2022a; Islam et al., 2023).

## 5 Transmission of MPXV

Some primates and rodents in central Africa serve as the natural Mpox hosts. Early MPXV infections took place owing to contact with infected animals, such as consumption of undercooked meat or exposure to tissues, body fluids, and mucous membranes. In addition, MPXV transmission can take place via bites or scratches from infected animals. Direct contact with respiratory droplets from infected persons is thought to be responsible for MPXV human-to-human transmission (Upadhayay et al., 2022; Beeson et al., 2023). Moreover, vertical transmission of MPXV can take place from infected mothers to their infants (Adler and Taggart, 2022; Billioux et al., 2022). Unlike previous Mpox outbreaks, the recent outbreak was the major reported Mpox outbreak outside of Africa. Previously, MPXV infection was only observed following traveling to Mpox-affected counties or after contact with infected animals (Kumar et al., 2023). Nonetheless, most of the confirmed Mpox cases of the current outbreak are not linked with travelling or direct contact with infected animals, but rather linked with sexual intercourse between individuals. Furthermore, it was observed that most of the Mpox cases were detected in bisexual or homosexual males. It has also been reported that 98% of the Mpox cases were observed among bisexual or homosexual males and 41% of them had co-infection with human immunodeficiency virus (HIV). Moreover, 73% of the lesions were observed in the genital and anal regions (del Rio and Malani, 2022). Substantial challenges for accurate diagnosis are faced because of this prolonged incubation period, which can further result in further MPXV transmission, disease progression, and delayed medical attention (Accordini et al., 2023; Lu et al., 2023; Reda et al., 2023).

## 6 Clinical presentation

Mpox infections can widely vary ranging from self-limiting and mild illness to a severe form of the disease in immunocompromised individuals with life-threatening conditions (Gessain et al., 2022; Mitjà et al., 2022; Titanji et al., 2022). The average MPXV incubation period ranges from 2 days to 3 weeks. On the other hand, the average incubation period during the clade IIb outbreak was found to range from 7-10 days (Miura et al., 2022; Tarín-Vicente et al., 2022). After an asymptomatic incubation period and before the appearance of a rash, Mpox patients usually have prodromal symptoms for 1-5 days including headache (25%-55%), malaise (23%-57%), myalgias (31%-55%), lymphadenopathy (56%-86%), and fever (62%-72%) (Patel et al., 2022; Philpott et al., 2022; Tarín-Vicente et al., 2022). Nonetheless, the timing of prodromal symptoms was found to be variable during the 2022 clade IIb outbreak, which arose after, before, or during the occurrence of the rash or other presenting symptoms (for example- proctitis) (Patel et al., 2022; Titanji et al., 2024). Painful skin lesion is the most common Mpox clinical feature that consistently advances through four distinct stages over two to four weeks. Typically, lesions start as macules and develop into pustules, vesicles, and papules, which desquamate and crust in the final stage. Individuals with Mpox remain infectious from the onset of clinical symptoms till all skin lesions are re-epithelialized, which usually takes up to four weeks. Mpox rash typically takes place in a centrifugal distribution, where lesions are observed in limbs, trunk, and face. Nonetheless, in the recent global outbreak, MPXV transmission predominantly occurred through sexual contact, which often resulted in anogenital and genital (36%-73%) as well as oral and perioral lesions (14%-25%), along with painful mucosal lesions at any site, limited (median number of 10) lesions, and nonuniform development of lesions (Patel et al., 2022; Thornhill et al., 2022; Titanji et al., 2024). Other complications including ocular disease, urethritis, pharyngitis (13%-36%), and proctitis (14%-36%) were also observed in Mpox individuals (Patel et al., 2022; Thornhill et al., 2022; Titanji et al., 2024). In addition, less than 5% of Mpox individuals show mucosal symptoms (pharyngitis or proctitis) without skin lesions or a single skin lesion without other symptoms. In healthcare workers, localized single lesions were reported at the site of a needle-stick injury (Choi et al., 2023; Titanji et al., 2024).

## 7 Mpox scenario in Saudi Arabia

In SA, the first laboratory-confirmed human Mpox case was reported on 14 July 2022 in Riyadh and a total of 8 confirmed cases as of 30 June 2024. Indeed, preventive measures are essential to prevent the MPXV transmission, which will further prevent an upcoming pandemic (Shoaib, 2023). In the beginning, there were no explicit guidelines for monitoring and regulating the transmission of MPXV in SA (Meo et al., 2022a). However, subsequently the Saudi Arabian MOH developed guidelines and implemented measures to control and monitor MPXV transmission in SA (Ajman et al., 2022). Saudi Arabian MOH contributed significantly in implementing and coordinating steps to regulate the COVID-19 pandemic in SA through international collaborations, vaccination campaigns, public communication, resource allocation, policy decisions, and influential leadership, which further resulted in decreased severe acute respiratory syndrome coronavirus 2 (SARS−CoV−2) transmission and eventually protected public health (Kamarudin et al., 2023; Hershan, 2024).

In a similar manner, Saudi Arabian MOH also has taken measures to control the spread of MPXV (Alshahrani et al., 2022a). If there is any suspected or confirmed Mpox case, it is more likely that MOH will take various preventive measures including contact tracing, isolation of the infected individuals, and monitoring of healthcare workers who might have come into the contact of MPXV. MOH has also conducted various public awareness campaigns to raise public awareness regarding the Mpox signs and symptoms in order to prevent Mpox transmission. Moreover, MOH has also considered offering the smallpox vaccines to high-risk populations, including healthcare professionals and people travelling to regions or countries where Mpox is endemic (Ajman et al., 2022). In a study in SA, Alshahrani et al (Alshahrani et al., 2022b). assessed the knowledge level of doctors about the Mpox. They reported that participant doctors exhibited highest knowledge level in clinical presentation 69.3% and disease transmission 70.4%. Nonetheless, the participants showed a lower level of knowledge (49.5%) in control and preventive measures. In addition, most of the participants showed positive attitudes toward controlling and preventing MPXV, where 84.1% of participants believed that with appropriate preventive measures, Mpox could be effectively controlled. Collectively, these findings suggested that it is essential to ameliorate the knowledge level of Saudi Arabian healthcare professionals regarding Mpox for effective control and prevention. Moreover, these measures not only will facilitate early identification and management of Mpox cases, but also the prevention of Mpox outbreaks (Shoaib, 2023).

In a different study, Meo et al (Meo et al., 2022a). assessed the attitudes, knowledge, awareness, public perception, and acceptance of immunization against pandemic threats including Mpox among 1020 study participants from Riyadh, SA. The researchers summarized that an increased number of study participants in favor of vaccination campaigns (62.8%) and implementation of preventive measures (43.7%) against Mpox. Therefore, improving public awareness and increased availability of information regarding Mpox are like to have significant contributions in strengthening the community to combat MPXV infections and lower the impact of Mpox in SA. Over 1.8 million pilgrims visited SA to perform Hajj and Umrah in 2023 alone, therefore it was challenging for responsible authorities to screen each traveler. Therefore, government health authorities of SA should strongly collaborate with various international health organizations including WHO to prevent, manage or monitor potential health risks at most of the entry points in SA including highways, seaports, and airports by ensuring adherence to hygiene protocols, vaccinations, and health screenings (Ajman et al., 2022; Shoaib, 2023).

## 8 Diagnostic testing for MPXV that can be used in Saudi Arabia

### 8.1 Real-time PCR

Any person satisfying the definition of a suspected case ought to be offered testing as per the guidelines of the U.S. CDC and WHO. In the case of lab-based diagnosis of Mpox, RT-PCR is regarded as the gold standard diagnostic method for samples derived from either wild animals or parents (Kulesh et al., 2004). A number of RT-PCR assays have been established for MPXV diagnosis after the emergence of Mpox. These RT-PCR methods have been developed for different targets in the MPXV genome including N3R, B6R, F3L, B7R, G2R, and TNF receptor gene. Clinical samples were used to evaluate the diagnostic validation. Most of the RT-PCR tests have a limit of detection (LoD) in the range of 250 to 10 copies per reaction (Shchelkunov et al., 2011). U.S. Food and Drug Administration (FDA) has granted emergency use authorization of 7 RT-PCR-based tests so far (da Silva et al., 2023).

### 8.2 Loop-mediated isothermal amplification

Although RT-PCR is regarded as the gold standard diagnostic method for Mpox, there are several drawbacks of these methods, such as the need for consistent access to electricity, technical expertise, long sample processing time, and usage of an advanced thermocycler for identification and amplification of MPXV genome (da Silva et al., 2019; Matthews et al., 2020; da Silva et al., 2021). Therefore, RT-PCR is inappropriate for dispersed applications, especially in middle- and low-income countries (da Silva et al., 2019). Easy-to-use as well as reliable assays and Point-of-Care Testing (POCT) are required to combat Mpox, particularly as the Mpox circulates in middle- and low-income countries. Considering all these issues, isothermal techniques including loop-mediated isothermal amplification (LAMP) might be the most outstanding methods for rapid Mpox detection (Da Silva et al., 2020a, 2020b). LAMP is a powerful, simple, low-cost, and rapid technique for rapid nucleic acid amplification at an isothermal and single temperature (for example- 60–65°C), which indicates that this method can be performed without the need for expensive equipment. Collectively, these features are extremely preferable for POCT usage in areas with limited laboratory facilities (da Silva et al., 2023).

### 8.3 Serological testing

When a test is carried out in isolation, it is advised to use serology for the clinical MPXV diagnosis to detect antibodies in serum or plasma (Gilchuk et al., 2016). The effectiveness of serological tests specific for MPXV is decreased via the potential for cross-reactivity with antibodies against other OPVs and those exhibited via vaccination, whether historical or recent. Therefore, it is indicated that serological testing should be limited to reference laboratories till further validation is given for the usage of serological or antibody-detecting POCTs outside such settings. When a serologically validated testing facility is available within a reference laboratory, the IgM detection in recently acutely ill individuals or IgG detection in paired serum specimens obtained at least 3 weeks apart from the initial sample collection during the first week of illness can enhance the accuracy of the diagnostic method, when results of other tests are inconclusive (Dubois et al., 2012). In the diagnostic framework, this technique highlights the importance of serology and its potential uses in certain conditions and within the limits of application standards and rigorous validation (Branda et al., 2024).

### 8.4 CRISPR/Cas-based systems

CRISPR-Cas systems are alternative emerging approaches of nucleic acid detection techniques that have simple device structure, high sensitivity and specificity, and outstanding compatibility with several methods for readout including fluorescence or lateral flow assays (LFAs) (Gootenberg et al., 2017, 2018; Broughton et al., 2020; De Puig et al., 2021; Kaminski et al., 2021). Extensive advancement has been achieved in the design of molecular diagnostic-based approaches by utilizing CRISPR/Cas components (Kaminski et al., 2021). The CRISPR/Cas systems detect viral nucleic acids based on their DNA or RNA sequences and then eradicate them by utilizing Cas enzyme-associated endonuclease function (Kaminski et al., 2021). The two groups of CRISPR systems were rapidly utilized for diagnostic purposes including DNA endonuclease-targeted CRISPR trans reporter (DETECTR) and specific high-sensitivity enzymatic reporter unlocking (SHERLOCK) (Gootenberg et al., 2018; Broughton et al., 2020; da Silva et al., 2023). Several studies already reported the potential of CRISPR-Cas systems in the detection of Mpox (Siegrist et al., 2020; Low et al., 2023; Zhao et al., 2023).

### 8.5 Whole-genome sequencing

WGS is a powerful, next-generation, and comprehensive sequencing technology that sequences the entire genome of an organism. WGS is considered as the most precise method in distinguishing MPXV from other OPVs, which provides extensive coverage of pathogens beyond other molecular techniques (Farlow et al., 2010). This method also promotes advanced virological study, allows comprehensive bioinformatic investigations, and the design as well as development of relevant immunoassays. WGS is a powerful method for detecting unknown transmission chains, specific genetic variants and strains, and possibly revealing the origin of an outbreak (Sharma et al., 2022; Vazquez et al., 2024). genetic evolution of MPXV can also be traced by analyzing WGS data, which can provide more data on MPXV adaptation across varied environments and hosts as well as detection of genetic markers for severe disease manifestations or antiviral resistance. Furthermore, WGS allows high-resolution mapping of MPXV biogeography and phylogeny and deducing patterns of the MPXV migration by comparing genome sequences derived from a range of outbreaks. There is a growing interest on WGS because of its potential in epidemiologic research, vaccine development, disease treatment, and development of targeted outbreak control and prevention approaches. Nonetheless, operational cost of WGS is high and it needs extensive computational resources, which restricts its uses in large-scale testing. There are several challenges in WGS applications, such as scientific, ethical, and practical considerations, as well as the requirement of thoughtful policymaking and constant development. Thus, WGS is not suitable as POCT because of these limitations. WGS mainly benefits specific case investigations and research initiatives as well as the creation of MPXV databases to enhance findings (Branda et al., 2024).

### 8.6 Electron microscopy

Electron microscopy can mediate the visual detection of potential MPXV within specimens. Nonetheless, because of the need for expensive research facilities, the requirement of specific technical expertise, and the emergence of more available molecular methods, this method is not widely used in MPXV diagnosis (Gentile and Gelderblom, 2005). Therefore, electron microscopy is not routinely used in the diagnosis of poxviruses (Branda et al., 2024).

### 8.7 Virus isolation and culture

Virus isolation and culture are time-consuming approaches that can be useful in diagnosing Mpox. This method can provide comprehensive characterization via sequencing, which can provide vital insights crucial for the developments of clinical applications, vaccines, research methodologies, and antiviral testing (Reed et al., 2004). MPXV isolation from important cases can mediate outbreak investigation and control measures via detecting the MPXV origin, reconstructing transmission chains, and pinpointing mutations via phenotypic and genomic comparisons among isolates. Indeed, MPXV shows vigorous growth in various mammalian cell lines including RK-13, BSC-1, Vero, and HeLa, as well as in chicken embryos (Erez et al., 2019). In chicken embryos, MPXV stimulates cytopathic activities in the chorioallantois membranes, which is more prominent in 1–4 days post-inoculation, including syncytium formation, cytoplasmic bridging, granulation, and cell rounding. On the other hand, typical detached and rounded cells become more noticeable in Vero cells within around 24 h, which allows identification of virus particles by specific antibodies and immunofluorescence. Notwithstanding the accuracy of this method, this method is not widely used because of the need for high-level biosafety labs (level 3 or higher), the risk of infection even after wearing adequate personal protection, the necessity for skilled personnel, and the extensive detection timeframe (Branda et al., 2024).

## 9 Prophylactic measures that can be taken in Saudi Arabia to prevent Mpox

### 9.1 Pre-exposure prophylaxis

#### 9.1.1 Individuals

People in SA might also play a role in limiting MPXV transmission in various ways. Confirmed or suspected individuals ought to stay home and avoid contact with other people. Immunocompetent people with mild Mpox symptoms ought to avoid contact with other people for 3 to 6 weeks (Suñer et al., 2023). In addition, healthy individuals ought to avoid intimate contact with Mpox-infected individuals. People should also maintain cough etiquettes, wear a fitted mask, practice good hand hygiene, and sneeze with a piece of cloth or tissue or by bending arms. Appropriate disinfection and cleaning of high-touch areas are suggested after having home visitors. Moreover, practicing safe sexual intercourse and having less sexual partners might help in limiting MPXV transmission. According to the CDC, around half of the individuals who are at greater risk have changed their sexual activities and the number of their sexual partners (Gupta et al., 2023).

#### 9.1.2 Surveillance systems and public awareness campaigns

**Figure 3:**
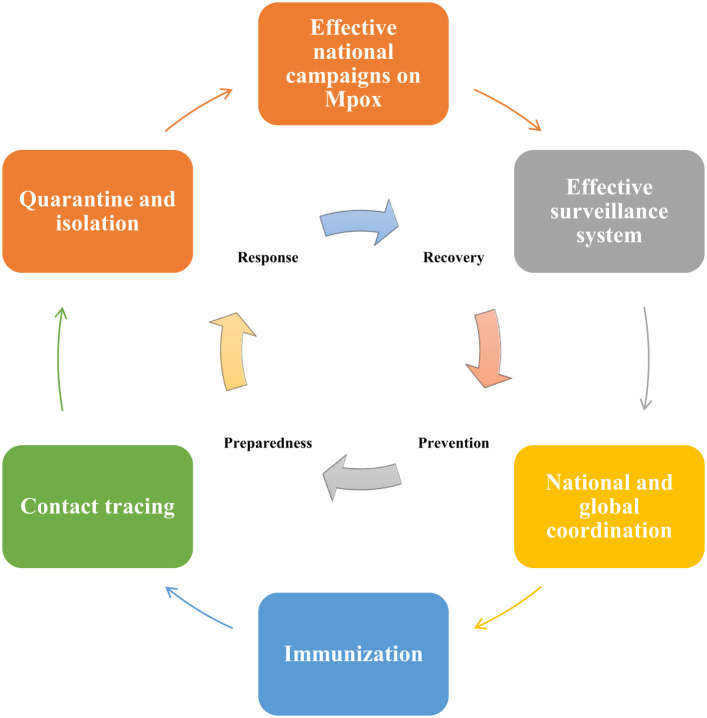
Pandemic preparedness and response plan to prevent Mpox outbreaks in Saudi Arabia (SA). The emergency management cycle of Mpox should follow 4 major phases including prevention, preparedness, response, and recovery. SA government can carry out public awareness campaigns to educate and raise awareness among the public regarding the signs and symptoms of Mpox. Suspected Mpox cases need to be reported and monitored by using the Health Electronic Surveillance Network of SA. Both local and global collaborations are essential for successful responses to Mpox-related public health emergencies. As there is no Mpox-specific vaccine available currently, therefore SA government can consider administering smallpox vaccines to high-risk groups, including healthcare professionals and individuals travelling to high-risk areas for Mpox. Contact tracing for Mpox is essential for the detection of new outbreaks and to stop or limit ongoing transmission. Quarantine and isolation of the infected individuals are crucial to minimize exposure to others.

Health Electronic Surveillance Network (HESN) is an electronic health information system of SA, that provides various public health data including immunization, vaccination, and epidemic outbreak (Alqifari et al., 2022). According to Ministry of Health (MOH) strict guidelines, any suspected Mpox cases must need to be reported by the healthcare facilities using HESN (Ministry of Health, 2023). This surveillance system ought to monitor suspected cases in SA and travelers from areas where Mpox is endemic (Shoaib, 2023). Confirmed or suspected Mpox cases ought to be isolated to avert the transmission of Mpox to others (**Figure 3**), this isolation is especially crucial in healthcare facilities where the risk of MPXV transmission is higher (Shoaib, 2023). Public awareness campaigns also need to be conducted by the SA government to educate the community regarding the Mpox signs and symptoms and methods to avert MPXV transmission (Shoaib, 2023).

#### 9.1.3 Healthcare workers

Mpox prevention in clinical settings is more challenging since the healthcare workers remain in contact with infected individuals (Guarner et al., 2022). Appropriate personal protective equipment (PPE) including gowns, masks, and gloves should be provided to healthcare workers to provide protection from Mpox exposure. Immediate masking of the confirmed or suspected individuals is also essential. Furthermore, skin lesions of the affected individuals also need to be covered with a cloth or gown and these individuals need to be isolated preferably in a negative-pressure-containing single-person room in the hospital. Workers should wear gloves while handling the laundry of Mpox-infected individuals (Guarner et al., 2022; Gupta et al., 2023).

#### 9.1.4 Vaccines

There is no Mpox-specific vaccine available currently. However, studies have reported that smallpox vaccines can provide up to 85% protection against Mpox (Kuroda et al., 2023; Liu et al., 2024). Moreover, epidemiological data suggest that increased occurrence of Mpox was observed in individuals who were born post-smallpox pandemic and eradication period or never been infected by poxviruses or never received smallpox vaccination in childhood (Malik et al., 2023a). At present, there are 2 approved smallpox vaccines used against Mpox. Pre- and post-prophylaxis immunizations for a certain group of people are recommended by various healthcare authorities including the Advisory Committee and Immunization Practices (ACIP) (Choudhary et al., 2022b; Meo et al., 2022b). FDA-approved vaccines including JYNNEOS™ and ACAM2000^®^ are recommended for healthcare professionals who are supposedly exposed to OPVs (**Table 1**) (Mahase, 2022; Payne et al., 2022). These healthcare professionals include scientists researching clinical Mpox samples, diagnostic and vaccination teams, response teams against outbreaks, clinical personnel associated with viral disease management, and laboratory workers as well as technicians. Therefore, proper vaccination guidelines should be developed in SA to ensure the safety of these healthcare professionals. In addition to the aforementioned 2 vaccines, Aventis Pasteur Smallpox Vaccine (APSV) is also approved for emergency uses when the two other vaccines are unavailable or contradicted for application. Smallpox vaccines were previously effectively used to prevent Mpox. As there is no report or literature currently available that shows the use of smallpox vaccine by the SA health authorities in Saudi Arabia, therefore SA government might offer smallpox vaccine to high-risk groups, including healthcare workers and individuals travelling to regions where Mpox is known to occur. Collectively, SA health authorities should continue to collaborate with international organizations including WHO to implement preventive strategies and measures to efficiently control the spread of MPXV in order to avoid the next Mpox pandemic (Shoaib, 2023).

**Table 1:** FDA-approved vaccines for Mpox prevention.

Vaccine	Vaccine type	FDA approval	Adverse reactions	Contraindications	References
ACAM 2000	Live vaccine	Approved on August 31, 2007 for a specific group of high-risk populations	Cutaneous reaction at the injection site	Severe immunodeficient individuals	(Gupta et al., 2023; Malik et al., 2023a)
JYNNEOS	Live attenuated vaccine	Approved on August 9, 2022 for the general population	No reaction at the injection site	Immunocompromised patients and individuals allergic to JYNNEOS vaccine	(Gupta et al., 2023; Malik et al., 2023a)

### 9.2 Post-exposure prophylaxis

Post-exposure prophylaxis is recommended for individuals following their contact with the skin or mucous membrane of an infected person or with their bedding, clothing, oral cavity, lesions, saliva, and bodily fluids (Mahase, 2022). People can be exposed to MPXV through viral presence in air particles and aerosol secretions, when they share a close space for a longer period (3 hours or more) (Islam et al., 2022c). Moreover, CDC and FDA recommend post-exposure vaccination only for high-degree exposures, where there is a possible risk of contracting MPXV (Islam et al., 2022c; Payne et al., 2022). Furthermore, the deficiency of medical masks and protective gloves or contact-used material without post- and pre-exposure sanitization is a condition that necessitates and sensitizes vaccination. It is recommended to go through monitoring or diagnosis before post-exposure prophylaxis, in cases of lower exposure rates or uncertain exposure (Bachmann and See, 2022; Islam et al., 2022c; Mahase, 2022). MPXV transmission can occur due to extended interaction with symptomatic individuals or infected animals. Therefore, according to CDC, post-exposure immunization should be carried out after around 4 days and within 4–14 days to prevent Mpox development (Bachmann and See, 2022; Payne et al., 2022). However, if vaccination is conducted after 2 weeks, only the disease burden can be reduced but the onset of Mpox cannot be averted (Bachmann and See, 2022; Raccagni et al., 2022; Saied et al., 2022b).

## 10 Therapeutics for Mpox treatment that can be used in Saudi Arabia

### 10.1 Antiviral agents

#### 10.1.1 Trifluridine

Trifluridine is a thymidine analog that acts as an antiviral drug by suppressing DNA synthesis. This antiviral drug suppresses DNA polymerase and also gets incorporated into DNA molecules, Nonetheless, trifluridine action might lack selectivity. In the case of Mpox, this drug is only used as a topical preparation on the eyes of infected individuals. Trifluridine does not cross the intact cornea, therefore it is considered safe when this drug is used as eye drops. Nonetheless, this drug might cross the cornea and be noticeable in aqueous humor in case of corneal pathologies disrupting its structure. Various mild adverse reactions have been reported with the use of trifluridine, including allergy, inflammation of the cornea, oedema of the eyelids, and temporary local burning sensation. The dosing interval of trifluridine is 2 hours until the complete regeneration of corneal epithelium. After the regeneration, it might be taken once every 4 hours for another 1 week. Nevertheless, trifluridine is not recommended to be used for a longer term. Alternative drugs might be selected if treatment is required beyond 3 weeks (Shamim et al., 2023). Cash-Goldwasser et al (Cash-Goldwasser et al., 2022). and Perzia et al (Perzia et al., 2022). carried out studies with trifluridine in the Mpox treatment. Nonetheless, this drug was used by the researchers in these studies as an add-on agent. In general, 5 participants received both tecovirimat and trifluridine (through topical route). Mpox clinical presentations of four participants varied greatly, but all of them exhibited ophthalmological manifestations. These 4 patients quickly recovered and were discharged. However, reduced visual acuity and worsened ocular symptoms were exhibited by one patient. Overall, no adverse events were observed with trifluridine (Cash-Goldwasser et al., 2022; Perzia et al., 2022).

#### 10.1.2 Brincidofovir

Brincidofovir is a prodrug of cidofovir that acts as an antiviral drug by selectively suppressing the activity of OPV DNA polymerase (**Figure 2**). Therefore, this drug was found to be efficient against DNA viruses. Brincidofovir is a lipid conjugate of cidofovir. It has been observed that lipid acylic nucleoside phosphonate plays a role as a phospholipid in the body and reduces the development of lesions with no confirmed effect with coupled vaccination (Hutson et al., 2021; McCarthy, 2022; Kuroda et al., 2023). Patients were reported to be completely recovered with minimal side effects. Therefore, this drug is approved to treat cytomegalovirus retinitis in HIV individuals and also approved to treat smallpox and other related infections. Brincidofovir is also a preferred drug for widespread administration during an emergency Mpox outbreak. As compared to cidofovir, brincidofovir has no reported nephrotoxicity and provides better oral bioavailability, thus brincidofovir received approval for oral administration (Hutson et al., 2021; McCarthy, 2022; Malik et al., 2023b). Nonetheless, the profile of liver enzymes needs to be strictly monitored and liver function tests need to be carried out for the treatment. Moreover, brincidofovir is not recommended for newborns, pregnant women, and immunocompromised individuals because of the hyperactive drug accumulation (Altindis et al., 2022). Animal studies already demonstrated its effectiveness against Mpox, however more clinical studies are required for proper licensure and approval (Bachmann and See, 2022; Malik et al., 2023a).

#### 10.1.3 Tecovirimat

Tecovirimat is an antiviral agent, which originally received FDA approval in 2018 to treat smallpox infection (Guarner et al., 2022). Tecovirimat can block the formation of the viral envelope by suppressing p37 polypeptide (**Figure 2**), which is a highly conserved sequence among all OPVs (Russo et al., 2021; Huang et al., 2022). As MPXV is an OPV, therefore tecovirimat might exhibit an antiviral role against Mpox. Mpox patients in the U.S. can obtain tecovirimat from state, county, or city health authorities through an FDA- and CDC-approved expanded access–investigational new drug protocol (Guarner et al., 2022; Gupta et al., 2023).

#### 10.1.4 Cidofovir

Cidofovir is effective against several DNA viruses and it acts as an antiviral drug by terminating DNA polymerase-based replication in the form of 5′-diphosphorylated metabolite (**Figure 2**). Cidofovir was found to be effective against infections caused by smallpox, Mpox (**Table 2**), VACV, and HIV (Al Jurdi and Kotton, 2022). Routes of administration of cidofovir include intravenous (IV) and topical routes. Indeed, cidofovir not only reduces the symptoms by preventing the formation of lesions, but also decreases mortality rates. In addition, cidofovir is used to treat severe VACV as a second-line therapy (Hutson et al., 2021; Malik et al., 2023b). Cidofovir was found to result in nephrotoxicity following IV administration, this drug should be administered along with probenecid and adequate hydration. Furthermore, proper dose adjustment along with renal functional considerations are needed to manage nephrotoxicity. Nonetheless, more clinical studies are required with cidofovir to confirm its potential against Mpox (Thakur, 2022; Malik et al., 2023a).

**Table 2:** A summary of the antivirals that can be used against Mpox.

Drug	Mechanism of action	Elimination half-life	Adverse reactions	Contraindications	References
Tecovirimat	Inhibits viral protein p37	18–26 hours	Vomiting, abdominal pain, nausea, and headache	Contraindicated in patients with creatinine clearance below 30 mL/min	(Bruno and Buccoliero, 2023; Gupta et al., 2023)
Brincidofovir	Inhibits DNA polymerase	19.3 hours	Abdominal pain, vomiting, nausea, and diarrhea	Contraindicated in women of childbearing potential	(Siegrist and Sassine, 2022; Gupta et al., 2023)
Trifluridine	Inhibits DNA polymerase	2.1 hours	neutropenia, leukopenia, anemia, and nausea	Contraindicated in individuals with a previous history of hypersensitivity to trifluridine	(Bruno and Buccoliero, 2023; Gupta et al., 2023)
Cidofovir	Inhibits DNA polymerase	3.2–4.4 hours	Asthenia, chills, fever, pancreatitis, proteinuria, rash, alopecia, diarrhea, vomiting, nausea, dyspnea, impaired hearing, iritis, headache, neutropenia	Contraindicated in individuals with cidofovir hypersensitivity, individuals taking nephrotoxic drugs, individuals with a urine protein ≥ 100 mg/dL, serum creatinine > 1.5 mg/dL, or creatinine clearance ≤ 55 mL/min	(Bruno and Buccoliero, 2023; Gupta et al., 2023)

### 10.2 Immunomodulators

Monoclonal antibodies (mAbs) are commonly used to treat orthopoxvirus infections, which can also be used to treat severe infections caused by MPXV (Xiao and Isaacs, 2010). It has been revealed that interferon beta can significantly suppress the Mpox generation and spread. Therefore, recombinant beta can be used as a safe and novel agent in Mpox treatment. More mAbs also need to be developed to elevate the potency of immunoglobulin therapy and/or specific antiviral drugs that may ameliorate outcomes of individuals with severe combined immunodeficient individuals (Johnston et al., 2012; Piparva et al., 2024).

### 10.3 Immunoglobulin therapy

In 2005, Vaccinia immunoglobulin IV (VIGIV) was first approved for clinical uses in treating complications associated with the use of smallpox vaccinations (Center for Infectious Disease Research and Policy, 2005; Piparva et al., 2024). Several recombinant immunoglobulins (rVIG) are currently being studied. rVIGs act in a strategic passive immunotherapy manner. VIG’s two IV formulations including VIGIV Dynport and VIGIV Cangene are being studied and yet to be licensed (O’Laughlin et al., 2022; Turner et al., 2022). It was previously utilized effectively in refractive cases. As a hyperimmune immunoglobulin, it plays a role in neutralizing virus particles and decreasing viremia and mortality rates by up to 30% to 40% (Payne et al., 2022). In order to develop passive immunity, antibodies are obtained from the plasma of smallpox-immune people. It is also used in individuals with immunodeficiency, since it has adequate maltose to perhaps raises revaccination needs, needs attention for renal insufficient profiles, might interfere with serological testing, and affect insulin levels and glycemic conditions (Bachmann and See, 2022). Since there is a lack of human trial data regarding Mpox treatment, therefore more studies are required in this regard (Malik et al., 2023a).

## 11 Challenges and future perspectives

Despite being a member of the DNA virus family, Mpox shows incredibly greater genomic variability because of elevated nucleotide polymorphism. Increased international travel and rapid population mobility have mediated the spread of Mpox among people, which is further facilitating its potential for mutation (Lu et al., 2023). In addition, these factors also play a role in drug resistance, increased variability, and appearance of multidrug-resistant strains of the Mpox virus (Kmiec and Kirchhoff, 2022). Future studies on Mpox in SA can result in a better understanding of Mpox and its potential to cause public health threats, which will also reduce knowledge gaps. A future Mpox pandemic might involve a serious burden in the case of economic development, social activities, education systems, and healthcare facilities. Although there is a deficiency of information regarding the specific impact of a Mpox pandemic, some findings indicated the need for assessment of the economic burden of Mpox treatment in healthcare settings, such as costs associated with hospitalization, public health interventions, medications, and diagnostics (Shoaib, 2023). Analysis of these factors in SA might aid the estimation of financial resources needed for effective Mpox outbreak management. Moreover, investigation is also required for the potential Mpox impact on the productivity of the workforce in various sectors (such as services, manufacturing, agriculture, and healthcare) because of reduced productivity, absenteeism, and illness.

## 12 Conclusions

In this review, important insights have been provided regarding epidemiology, transmissions, clinical presentation, diagnostic tests, prophylactic measures and therapeutic options of Mpox from SA perspective. Recent Mpox outbreaks around the world have emphasized the necessity for vigilant and constant monitoring in SA. Mpox cases in SA were found to be travel-associated and showed potential close contact and heterosexual transmission. Novel therapeutic and prophylactic measures are also needed to be continuously appraised for the potential implications in SA. Selective usage of smallpox vaccines in the SA population can also be considered to limit MPXV spread. Moreover, lessons learned from the Covid-19 pandemic should be applied in the containment and management of Mpox in SA.
